# The significance of refuge heterogeneity for lowland stream caddisfly larvae to escape from drift

**DOI:** 10.1038/s41598-019-38677-6

**Published:** 2019-02-14

**Authors:** J. H. F. de Brouwer, M. H. S. Kraak, A. A. Besse-Lototskaya, P. F. M. Verdonschot

**Affiliations:** 1 Alterra, Wageningen University and Research, Department of Freshwater Ecology, P.O. Box 47, 6700 AA Wageningen, The Netherlands; 2 University of Amsterdam, Institute for Biodiversity and Ecosystem Dynamics (IBED), P.O. Box 94248, 1090 GE Amsterdam, The Netherlands

## Abstract

The process of macroinvertebrate drift in freshwater lowland streams is characterized by dislodgement, drift distance and subsequent return to the bottom. Refuges are important to all drift phases, since they may help larvae to avoid dislodgement and to escape from drift, even more so if the refuge structure is complex and heterogeneous. The aim of the present study was therefore to determine the influence of refuge heterogeneity on the ability of caddisfly larvae to return to the bottom from drift and to avoid secondary dislodgement. To this purpose a series of indoor flume experiments were undertaken, testing six Limnephilidae (Trichoptera) species, that occur on a gradient from lotic to lentic environments. Bed morphology (plain, refuges with or without leaf patches) and flow velocity (low (0.1 m/s), intermediate (0.3 m/s) and high (0.5 m/s) were manipulated. We showed that all species were favoured by refuges and that especially for species on the lentic end of the gradient (*L*. *lunatus*, *L*. *rhombicus* and *A*. *nervosa*), the ability to escape from drift and to avoid secondary dislodgement was increased. Moreover, we showed that all species spent more time in refuges than in open channel parts and more time in heterogeneous refuges (leaf patches) than in bare refuges, the latter being especially the case for larvae of the lotic species. For lentic species, not well adapted to high flow velocity, refuges are thus crucial to escape from drift, while for the lotic species, better adapted to high flow velocity, the structure of the refuge becomes increasingly important. It is concluded that refuges may play a crucial role in restoring and maintaining biodiversity in widened, channelized and flashy lowland streams.

## Introduction

Drift is regarded as the dominant form of macroinvertebrate movement in freshwater lowland streams^1,2^, travelling short to long distances before returning to the stream bottom^3,4^. Previous studies revealed that drift densities of most species increase with increasing flow velocity^5–8^, but drifting invertebrates will eventually need to escape from the water column to prevent being washed out of the system. Hence, the process of drift is characterized by dislodgement, drift distance and subsequent return to the bottom^9^. The fate of most dislodged organisms however, is poorly understood^9–11^ and abilities of invertebrates to use behavioural moves to end drifting are scarcely documented^12,13^. Previously we studied the ability of six Trichoptera species ranging from lentic to lotic, to return to the stream bottom under different flow velocity conditions^14^. A gradient of flow velocity tolerance and species specific abilities to escape from drift was observed, indicating that, in addition to dislodgement, the process of returning to the bottom is of equal importance in determining flow velocity tolerance of Trichoptera species^14^.

All phases of the drift process, dislodgement, drift distance and return to the bottom, may highly depend on the heterogeneity of the habitat, since structures and substrates can ameliorate negative effects of flow disturbance on benthic invertebrates^15–18^. Heterogeneous environments, that include stable habitat patches like leaf packages, offer refuges in which organisms can find shelter. This may help individuals to avoid dislodgement, since movement to refuges prior to high flow is a commonly used avoidance strategy to prevent dislodgement^19–21^. Refuges may also serve as focal points for individuals increasing return rates to the bottom from drift, but this beneficial role of refuges in ending drift has only been scarcely documented. Once returned to the bottom, resilience and resistance traits, like a streamlined, flattened small body and possessing means to cling to the substratum^22,23^, that enabled the different species to return^14^, may also help them to prevent secondary dislodgement, and even more so if the refuge structure is complex and heterogeneous. This, however, has never been studied. The aim of the present study was therefore to determine the influence of refuge heterogeneity on the ability of caddisfly larvae to return to the bottom from drift and to avoid secondary dislodgement. To this purpose a series of indoor flume experiments were undertaken, testing six Limnephilidae (Trichoptera) species, that occur on a gradient from lotic to lentic environments^14,24^. Bed morphology and flow velocity were manipulated. We hypothesized that the presence of refuges in streams increases return rates to the bottom from drift, helps to avoid secondary dislodgement and that heterogeneous refuges (leaf patches) are used more effectively by caddisfly larvae than bare refuges.

## Materials and Methods

### Test species

The Limnephilidae are a relatively large family compromising many species with large differences in ecology and distribution, despite a high morphological similarity. Six species of Limnephilidae were selected for this experiment: *Limnephilus lunatus* (Curtis, 1834), *Limnephilus rhombicus* (Linnaeus, 1758), *Anabolia nervosa* (Curtis, 1834), *Halesus radiatus* (Curtis, 1834), *Chaetopteryx villosa* (Fabricius, 1798) and *Micropterna sequax* (McLachlan, 1875). The selected species occur in North-West European sandy lowland streams in respective order along a gradient from lentic to lotic environments^25–27^. Within lowland streams, the first three species can be considered lentic and the latter three lotic^24^, responding clearly differently to flow velocity^14^.

Approximately 1500 fifth instar larvae were collected and all indivduals were identified one by one. They were manually picked from sites where large populations of the respective species occur. Specimens were collected from the Warnsbornse beek, Coldenhovense beek, Seelbeek and drainage ditches (the Netherlands). Specimens were kept in an artificial rearing-stream in separate compartments containing 200–300 conspecifics and a surplus of organic material (detritus, leaves, twigs and plants) on a bottom of fine gravel and sand. Food levels were kept high by adding extra leaves, detritus and wheat fragments weekly. Environmental conditions in the laboratory rearing-stream were kept constant with a water temperature of 10 °C, a flow velocity range of 0.05–0.10 m/s and a day:night light regime of 16:8 h, reflecting a natural spring setting.

### Outline of the study

To determine the influence of refuges with or without leaf patches on the ability of caddisfly larvae to return to the bottom from drift and to avoid secondary dislodgement, a series of indoor flume experiments were undertaken with the six selected Limnephilidae species. The responses of the test species to three different flow velocities were tested in channels with plain beds (control) and with refuges with or without leaf packages (treatments).

### Experimental flumes

**Figure 1:**
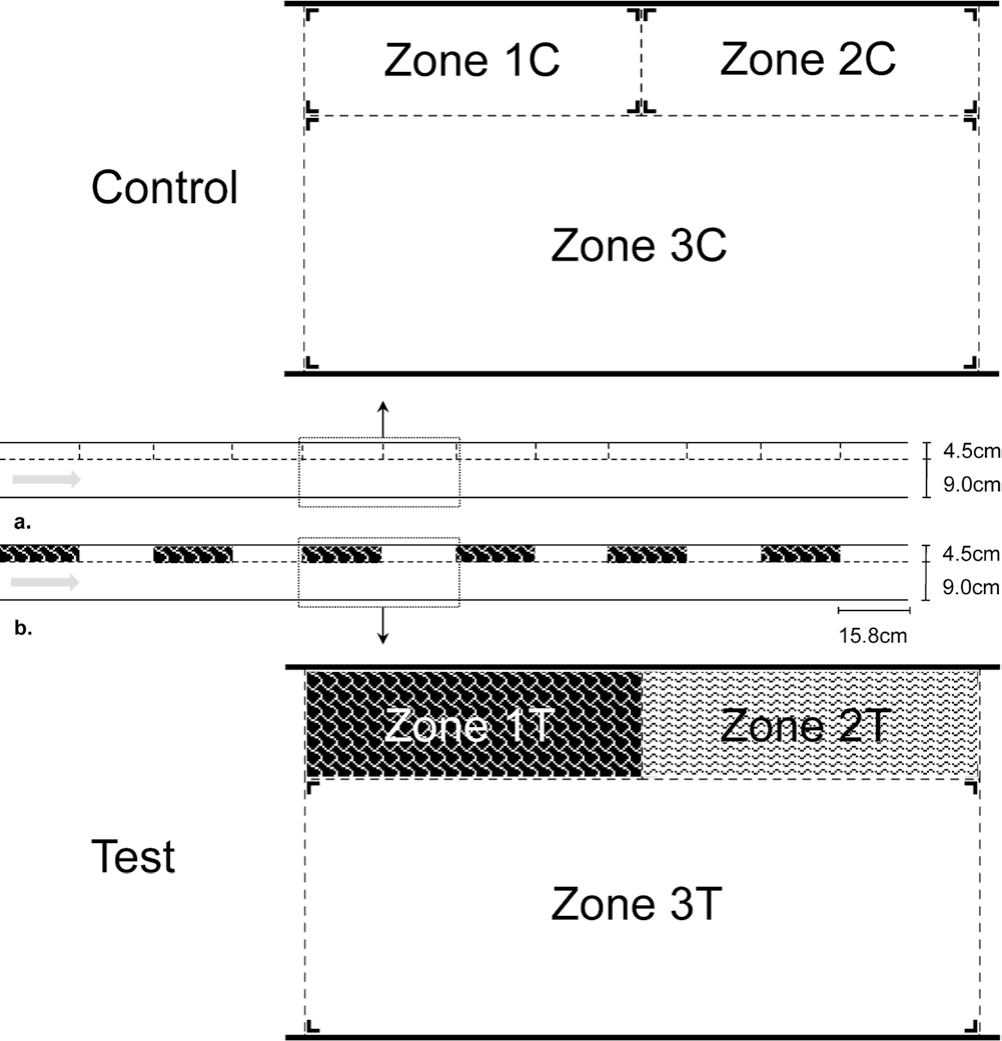
Schematic overview of the control (**a**) and the test (**b**) channel. The control channel consisted of a planar bottom habitat in Zone 1 C, Zone 2 C and Zone 3 C. The test channel included leaf patches (black shaded area) in Zone 1T, bare refuges (white shaded area) in Zone 2T and a planar bottom habitat (white area) in zone 3T. Leaf patches and bare refuges occurred six times on the longitudinal axis of the channel.

The experiments were conducted in a channel (Fig. 1), which is part of a fully controlled recirculating laboratory flume system with adjustable flow velocity. Water is stored in a reservoir from which it is pumped through flow-homogenizing lamellae to flow through the channel before returning to the reservoir^28^.

### Bed morphology

The stream bed of the channel was comprised of sand grains (<250 µm) glued to acrylic plates, mimicking a flat, sandy stream bottom. A control channel and a test channel were established (Fig. 1). The test channel consisted of three zones: Zone 3T, two-thirds of the width of the channel, represented a bare homogeneous bottom morphology. The zones 1T and 2T, one-third of the width of the channel, consisted of 12 alternating refuges, with or without leaf packages. The leaf packages consisted of *Quercus rubor* leaves attached to frames, which were fixed to the channel bed, such that macroinvertebrates could enter and leave these patches (Fig. 1b). The control channel consisted entirely of a bare homogeneous bottom, but to compare the control and the treatment channel, we referred to the same spatial zones as in the treatment channel (Zone 1 C, Zone 2 C and Zone 3 C)(Fig. 1a).

### Flow velocity

All tests (6 species x 20 specimens x 3 flow velocities = 360 runs) were conducted under constant water temperature and light regime. Three flow velocities were tested: low (0.1 m/s), intermediate (0.3 m/s) and high (0.5 m/s), reflecting the natural range of flow conditions in lowland streams^29^. Near bed flow velocity was measured in the water column above all zones in all treatments, at the centre of the channel and in the refuges, using an electromagnetic flow meter (SENSA RC2 ADS, model V6d).

### Experimental runs

Per test run, one specimen was released in the water column at the entrance of the test section and monitored while the flow velocity was kept constant. Test specimens were free to move upstream and downstream after release in the test section for a maximum of 6 minutes. Preliminary tests showed that 6 minutes were suited to ensure that specimens attached firmly and to rule out secondary dislodgements. We tested twenty different specimens (replicates) per species per flow velocity treatment. Experiments were stopped if specimens reached the lower end of the test section within the 6 minutes, which were then classified as ‘lost by drift’. The time individuals remained in the experiment was used as an indicator for: (1) the ability to return to the bottom from drift and (2) the resistance to secondary dislodgement. For the animals that remained the entire 6 minutes test period in the channels, we continuously monitored the time individuals spent in the different spatial zones (Fig. 1), using visual observations. These visual observations were inputted into computer software, registering the time spent in the different habitat categories, bare stream sediment and refuges with or without leaf packages (Noldus, Observer®XT 10.5).

### Data Analysis

To evaluate if the experimental design resulted in the desired differences in flow velocities between the bare homogeneous bottom and refuges with or without leaf packages, one way ANOVA applying a Bonferroni correction was used. Next, we assessed whether there were significant differences in residence time of caddisfly larvae between the entire control channel (Fig. 1a: 1 C + 2 C + 3 C) and the test channel (Fig. 1b: 1T + 2T + 3T) using Mann-Whitney U tests. To quantify the attractiveness of refuges, we compared the time spent in Zone 1T + 2T by individuals that returned to the bottom with the time spent in 1 C + 2 C, using a Mann-Whitney U test. Subsequently, we assessed whether individuals merely seek refuges or specifically the physical structures offered by the heterogeneous refuges (leaf patches) by comparing the time individuals spent in Zone 1T (leaf patches) and in Zone 2T (bare refuges) using Wilcoxon Signed Rank tests.

In addition to subjecting the data to traditional statistics, we employed an intergrated modeling approach allowing to analyse all data simultaneously and, most importantly, to identify interactions between the experimental variables. To this purpose we selected a parsimonious model on the basis of having the lowest corrected Akaike Information Criterion (AICc) value using the dredge function in the R package MuMin version 1.15.6^30^ and report the two best models and their main and interaction effects. Statistical analysis was carried out in R (version 3.0).

## Results

**Figure 2:**
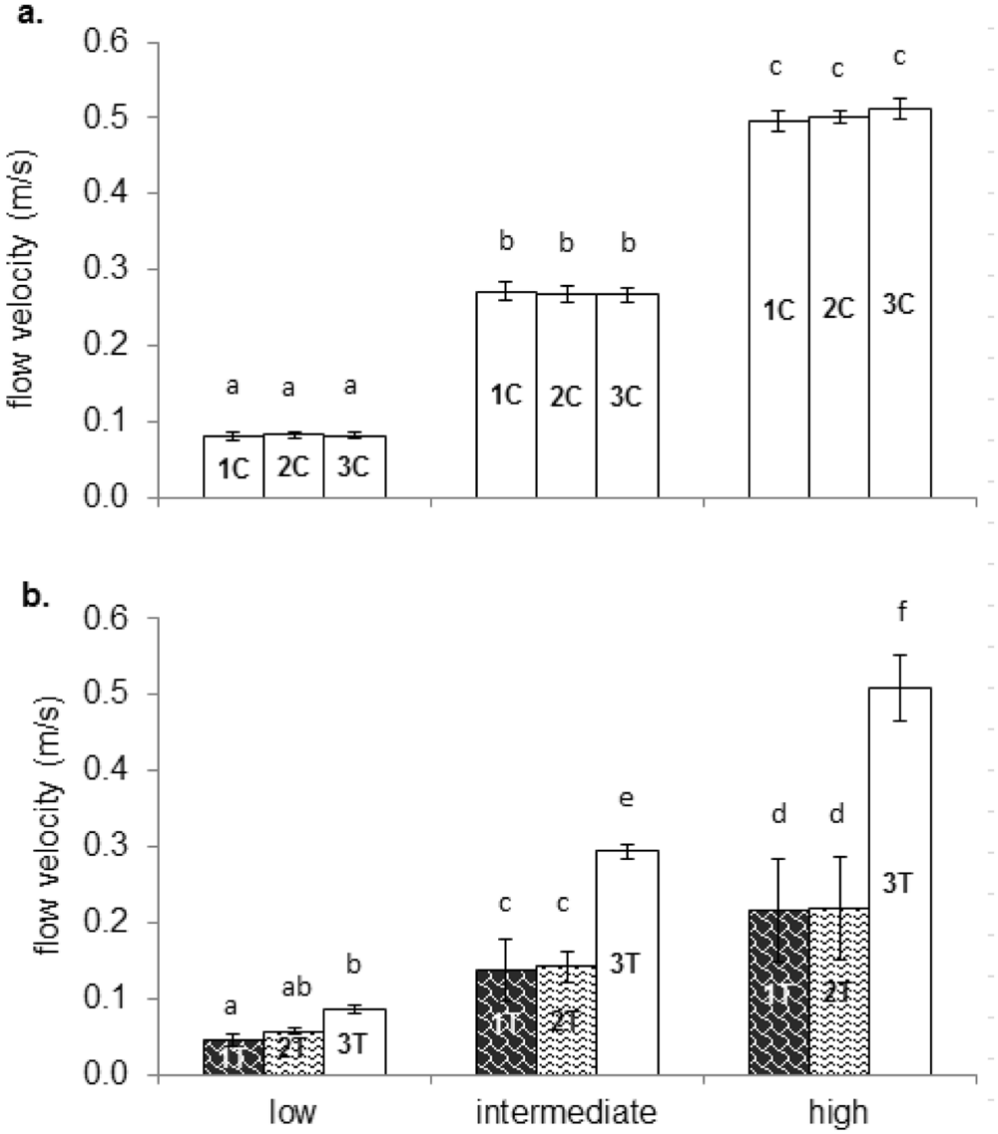
Mean (±SD) flow velocity in the control (**a**) and test (**b**) channel per flow velocity (low (0.1 m/s), intermediate (0.3 m/s) and high (0.5 m/s)). Leaf patches (black shaded bars), bare refuges (white shaded bars) and planar bottom habitat (white bars) are distinguished. Central codes in the bars indicate the channel zone. Different letters above the bars indicate significant differences (Bonferroni test, *P* < 0.05).

In the control channel, flow velocities were not significantly different between Zone 1 C, Zone 2 C and Zone 3 C (Fig. 2). In the test channel, flow velocities in Zone 1T (leaf patches) and Zone 2T (bare refuges) were similar and approximately 50% of those in the open channel (Zone 3T). Moreover, flow velocities showed no significant differences between the control channel (Zone 3 C) and the open part of the treatment channel (Zone 3T) and matched the targeted values.

**Figure 3:**
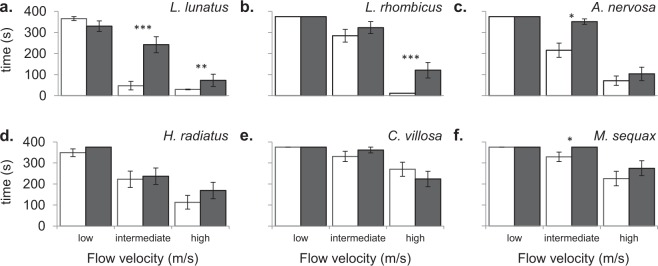
Mean (±SD) time *L*. *lunatus* (**a**), *L*. *rhombicus* (**b**), *A*. *nervosa* (**c**), *H*. *radiatus* (**d**), *C*. *villosa* (**e**) and *M*. *sequax* (**f**) individuals spent in the control (white bars) and test channel (black bars) per flow velocity (low (0.1 m/s), intermediate (0.3 m/s) and high (0.5 m/s)). Asterisks indicate significant differences between the control and the test channel (*P = 0.05–0.01, **P = 0.01–0.001, ***P = 0.001–0.001). The maximal test duration was 360 seconds.

The higher the flow velocity, the fewer individuals remained in the channels (Table 1) and the shorter the time they remained in the channels (Fig. 3). Yet, species and treatment specific differences were also observed. The number of individuals remaining in the channels and the residence time increased over the lentic-lotic species gradient, with *L*. *lunatus* being the most vulnerable to high flow velocity, in contrast to *C*. *villosa* and *M*. *sequax* (Table 1; Fig. 3). At intermediate and high flow velocity, in the test channel (1T + 2T + 3T) a significant (P < 0.05) higher number of individuals returned to the bottom from drift and remained in the system than in the control channel (1 C + 2 C + 3 C) (Table 1). Moreover, all species remained longer in the test channel (1T + 2T + 3T) than in the control channel (1 C + 2 C + 3 C) and in five cases this difference was significant (P < 0.05), especially concerning the lentic species (four out of five cases; Fig. 3).

**Table 1:** Number of individuals (out of 20) that escaped from drift and remained in the channels during the 360 seconds test period.

Species		control (C)	test (T)
*L*. *lunatus*	low	19	17
*L*. *lunatus*	intermediate	1	12
*L*. *lunatus*	high	0	3
*L*. *rhombicus*	low	20	20
*L*. *rhombicus*	intermediate	13	17
*L*. *rhombicus*	high	0	5
*A*. *nervosa*	low	20	20
*A*. *nervosa*	intermediate	8	16
*A*. *nervosa*	high	1	4
*H*. *radiatus*	low	17	20
*H*. *radiatus*	intermediate	10	12
*H*. *radiatus*	high	3	8
*C*. *villosa*	low	20	20
*C*. *villosa*	intermediate	16	19
*C*. *villosa*	high	12	10
*M*. *sequax*	low	20	20
*M*. *sequax*	intermediate	15	20
*M*. *sequax*	high	10	14

**Figure 4:**
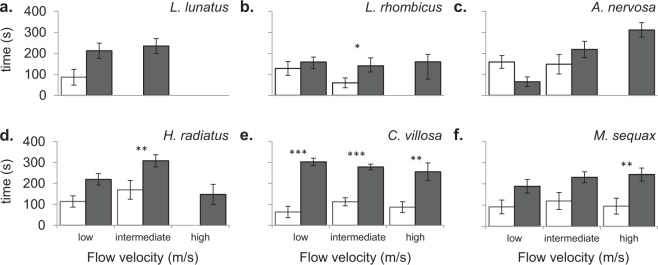
Mean (±SD) time *L*. *lunatus* (**a**), *L*. *rhombicus* (**b**), *A*. *nervosa* (**c**), *H*. *radiatus* (**d**), *C*. *villosa* (**e**) and *M*. *sequax* (**f**) individuals spent in the control zones 1 C + 2 C (white bars) and in the refuges 1T + 2T (black bars) per flow velocity (low (0.1 m/s), intermediate (0.3 m/s) and high (0.5 m/s)). Asterisks indicate significant differences between the control zones 1 C + 2 C and the refuges 1T + 2T (*P = 0.05–0.01, **P = 0.01–0.001, ***P = 0.001–0.001). Only data for the individuals that remained in the experiment for the entire 360 seconds test period are included.

With only a single exception (*A*. *nervosa* at low flow velocity), all species spent more time in the refuges (with or without leaves; 1T + 2T) than on the bare homogeneous bottom (1 C + 2 C) (Fig. 4). In six cases this difference was significant (P < 0.05), five cases concerning lotic species. In four other cases (three lentic and one lotic species) no time at all was spent on the bare homogeneous bottom (1 C + 2 C), while considerable time was spent in the refuges (with or without leaves; 1T + 2T) (Fig. 4).

**Figure 5:**
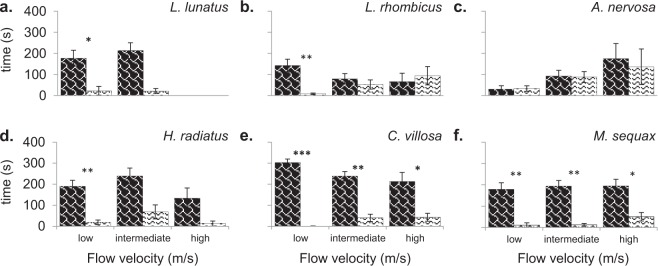
Mean (±SD) time *L*. *lunatus* (**a**), *L*. *rhombicus* (**b**), *A*. *nervosa* (**c**), *H*. *radiatus* (**d**), *C*. *villosa* (**e**) and *M*. *sequax* (**f**) individuals spent in the leaf patches (black shaded bars) and in the bare refuges (white shaded bars) per flow velocity (low (0.1 m/s), intermediate (0.3 m/s) and high (0.5 m/s)). Asterisks indicate significant differences between the leaf patches (1T) and the bare refuges (2T) (*P = 0.05–0.01, **P = 0.01–0.001, ***P = 0.001–0.001). Only data for the individuals that remained in the experiment for the entire 360 seconds test period are included.

Irrespective of flow velocity, in fourteen out of seventeen cases the larvae spent more time in leaf patches (1T) than in the bare refuges (2T) (Fig. 5). In nine cases this difference was significant (P < 0.05), especially concerning the lotic species (seven out of nine cases; Fig. 5).

The parsimonious logistic regression model (Supplemental Table 1) selected on the basis of AICc showed that the probability of escaping from drift was higher in the test channel than in the control channel, was higher for lotic species than for lentic species and decreased with increasing flow velocity. The model contained one interaction term (P < 0.05), between type of species and flow velocity, showing that the difference in probability of escaping from drift between lotic species and lentic species increased with increasing flow rate.

## Discussion

The process of drift is characterized by dislodgement, drift distance and subsequent return to the bottom^9^. While dislodgement is well studied, the fate of drifting organisms is poorly understood. In a previous study^14^ we therefore determined the ability of six Trichoptera species to return to the stream bottom under different flow velocity conditions and demonstrated that species on the lotic end of the gradient had highest return rates at high flow velocity and used active behaviour most efficiently to return to the bottom from drift. Subsequently, in the present study we aimed to elucidate the importance of refuge heterogeneity for the same six caddisfly species to escape from drift and to avoid secondary dislodgement. We showed that all species benefitted from refuges and that especially for species on the lentic end of the gradient (*L*. *lunatus*, *L*. *rhombicus* and *A*. *nervosa*), the ability to escape from drift and to avoid secondary dislodgement was increased. Moreover, all species spent more time in refuges than in open channel parts and more time in heterogeneous refuges (leaf patches) than in bare refuges, the latter being especially the case for larvae of the lotic species.

Our results thus suggest that the characteristics of the refuge are important. Except for *A*. *nervosa*, all species spent more time in heterogeneous refuges (leaf patches) than in bare refuges, indicating the higher importance of habitat structure over merely low flow, especially for the lotic species. Meanwhile, comparing the number of larvae that were able to escape from drift between the control and test channel revealed that, although beneficial to all species, the differences were largest for the lentic species. Hence it is concluded that for the lentic species, not well adapted to high flow velocity, refuges are crucial to escape from drift. The lentic species seek refuges to escape flow, more independent being it leaves or bare refuges. While for the lotic species, better adapted to high flow velocity, the structure of the refuge becomes increasingly important, as refuges also provide structure and food. Being able to cope with flow, their preference may be more food driven. In agreement, Verdonschot *et al*.^28^ reported that these species indeed show a strong preference for leaf habitats.

Several studies showed that local hydromorphological conditions influence settlement rates^31–34^. Some studies even reported larval movements to sites where hydraulic forcing is relatively low prior to extreme events to evade floods^10,35–37^, which is especially effective if disturbance events are predictable^37–39^. Also Oldmeadow *et al*.^13^ showed that two Ephemeroptera species actively swam towards low flow areas while in drift, but also that both species differed in their ability to reach those refuges.

The role of refuges in avoiding and overcoming the adverse effects of floods depends on the stability of the refuges. Hauer *et al*.^40^ considered habitat stability to be a morphodynamic necessity for aquatic organisms in their study on fish spawning, and for macroinvertebrates, substrate erosion can indeed induce catastrophic drift^41^. This may imply that escaping from drift is more likely in streams where refuges are stable and abundant. Such streams may better sustain macroinvertebrate communities, because the recovery of a community from high drift loss depends on new colonists^42^, originating from refuges. This becomes even more important in the nowadays often widened, channelized and flashy lowland streams where the studied species occur, and where flow velocities frequently exceed 0.3 m/s and even 0.5 m/s^43,44^. Based on the results of the present study we therefore argue that especially the more lentic and littoral species, *L*. *lunatus*, *L*. *rhombicus* and *A*. *nervosa*^25,26^, may only thrive in channelized lowland streams if refuges are abundantly present to limit population depletions during high flows.

This study highlighted the importance of refuges in freshwater lowland streams for caddisfly larvae to escape from drift and to avoid secondary dislodgement. Active movements in drift and the ability to move into refuges are key strategies to minimise drift and hence displacement distance. Flow regimes of many streams have, however, become more flashy and unpredictable^45–47^ by increasing drainage infrastructure and maintenance activities to enhance run-off from urban and agricultural area’s^44,48,49^. Channelization and maintenance measures have reduced the number of refuges, such as leaf patches, organic debris and wood, while vegetation is often periodically removed^50–53^. Hence, lowland streams have changed into multi-stress environments and communities in channelized sections of streams are less persistent than those in natural sections with refuges^54^ and refuges increase community persistence during high flows^55^. Refuges may thus play a crucial role in restoring and maintaining biodiversity in widened, channelized and flashy lowland streams.

## Supplementary information

Supporting info
