# Supplementary material for: The significance of refuge heterogeneity for lowland stream caddisfly larvae to escape from drift
Source: Sci Rep. 2019 Feb 14;9:2140. doi: 10.1038/s41598-019-38677-6 (PMC6375960; doi:10.1038/s41598-019-38677-6)
Supplement: Supplementary file 1 — Supporting info [file 41598_2019_38677_MOESM1_ESM.doc]

**The significance of refuge heterogeneity for lowland stream caddisfly larvae to escape from drift**

J.H.F. de Brouwer^1^, M.H.S. Kraak^2*^, A.A. Besse-Lototskaya^1^, P.F.M. Verdonschot^1,2^.

Supplementary Information

**Supplemental Table I**

# Table1Refuges.r

braak008

Tue Oct 11 11:40:33 2016

rm(list=ls(all=TRUE))
#setwd("D:/Data/Aa/_projects/JandeBrouwer")
Table1Refuges <- read.csv("Table1Refuges.csv")
library(reshape2)
# Specify id.vars: the variables to keep but not split apart on
Data = melt(Table1Refuges, id.vars=c("Species", "Flow"), value.name = "N_remain", variable.name = "ChannelType")
Data$N_float = 20 - Data$N_remain


levels(Data$Species) #"A. nervosa" "C. villosa" "H. radiatus" "L. lunatus"

## [1] "A. nervosa" "C. villosa" "H. radiatus" "L. lunatus"
## [5] "L. rhombicus" "M. sequax"

#[5] "L. rhombicus" "M. sequax"
#c("lentic","lotic")
Data$SpGr = factor(c("lentic","lotic","lotic","lentic","lentic","lotic")[Data$Species])

Data$Flow = factor(Data$Flow,c("low","intermediate","high"))

# For logistic regression in R see:
# #https://ww2.coastal.edu/kingw/statistics/R-tutorials/logistic.html
# #> glm.out = glm(cbind(Menarche, Total-Menarche) ~ Age, family=binomial(logit), data=menarche)
# #for our data:
# summary(glm(cbind(N_remain, N_float)~ChannelType,family= binomial, data = Data))
# gives a positive coefficient for ChannelTypeTest, as number that remain in Test is higher than in Control (257 vs 205 with fixed total)
# with(Data, sum(N_remain[ChannelType == "Control"]))
# with(Data, sum(N_remain[ChannelType == "Test"]))

library(MuMIn) # for model selection in R

glm0 = glm(cbind(N_remain, N_float)~SpGr*Flow*ChannelType,family= binomial, data = Data, na.action =na.fail)
drMod = dredge(glm0)

## Fixed term is "(Intercept)"

drMod

## Global model call: glm(formula = cbind(N_remain, N_float) ~ SpGr * Flow * ChannelType,
## family = binomial, data = Data, na.action = na.fail)
## ---
## Model selection table
## (Int) ChT Flw SpG ChT:Flw ChT:SpG Flw:SpG ChT:Flw:SpG df logLik
## 40 2.9560 + + + + 7 -80.588
## 56 2.8550 + + + + + 8 -78.926
## 8 2.6090 + + + 5 -84.563
## 24 2.5280 + + + + 6 -83.909
## 48 3.2290 + + + + + 9 -79.490
## 16 2.8740 + + + + 7 -83.063
## 128 4.0780 + + + + + + + 12 -73.362
## 64 3.1000 + + + + + + 10 -78.174
## 32 2.8350 + + + + + 8 -82.587
## 39 3.3670 + + + 6 -94.453
## 7 3.0330 + + 4 -98.213
## 4 3.1260 + + 4 -108.635
## 12 3.3670 + + + 6 -107.365
## 3 3.5050 + 3 -121.106
## 6 -0.1483 + + 3 -237.404
## 22 -0.1782 + + + 4 -237.309
## 5 0.1782 + 2 -245.972
## 2 0.2796 + 2 -252.703
## 1 0.5826 1 -260.912
## AICc delta weight
## 40 179.2 0.00 0.348
## 56 179.2 0.01 0.346
## 8 181.1 1.95 0.131
## 24 182.7 3.54 0.059
## 48 183.9 4.73 0.033
## 16 184.1 4.95 0.029
## 128 184.3 5.11 0.027
## 64 185.1 5.97 0.018
## 32 186.5 7.33 0.009
## 39 203.8 24.63 0.000
## 7 205.7 26.54 0.000
## 4 226.6 47.38 0.000
## 12 229.6 50.45 0.000
## 3 249.0 69.78 0.000
## 6 481.6 302.38 0.000
## 22 483.9 304.73 0.000
## 5 496.3 317.13 0.000
## 2 509.8 330.59 0.000
## 1 523.9 344.76 0.000
## Models ranked by AICc(x)

glm1 = glm(cbind(N_remain, N_float)~ChannelType+Flow+SpGr+ Flow:SpGr,family= binomial, data = Data, na.action =na.fail)
glm2 = glm(cbind(N_remain, N_float)~ChannelType+Flow+SpGr+ Flow:SpGr + ChannelType:SpGr,family= binomial, data = Data, na.action =na.fail)


summary(glm2)

##
## Call:
## glm(formula = cbind(N_remain, N_float) ~ ChannelType + Flow +
## SpGr + Flow:SpGr + ChannelType:SpGr, family = binomial, data = Data,
## na.action = na.fail)
##
## Deviance Residuals:
## Min 1Q Median 3Q Max
## -3.4706 -1.3285 0.6716 1.1719 2.7101
##
## Coefficients:
## Estimate Std. Error z value Pr(>|z|)
## (Intercept) 2.8546 0.5160 5.532 3.17e-08 ***
## ChannelTypeTest 1.5376 0.3358 4.579 4.66e-06 ***
## Flowintermediate -3.3529 0.5557 -6.033 1.61e-09 ***
## Flowhigh -5.9532 0.6255 -9.518 < 2e-16 ***
## SpGrlotic 0.4997 0.7859 0.636 0.5249
## Flowintermediate:SpGrlotic 0.8510 0.8366 1.017 0.3090
## Flowhigh:SpGrlotic 2.1206 0.8782 2.415 0.0158 *
## ChannelTypeTest:SpGrlotic -0.7884 0.4362 -1.807 0.0707 .
## ---
## Signif. codes: 0 '***' 0.001 '**' 0.01 '*' 0.05 '.' 0.1 ' ' 1
##
## (Dispersion parameter for binomial family taken to be 1)
##
## Null deviance: 450.08 on 35 degrees of freedom
## Residual deviance: 86.11 on 28 degrees of freedom
## AIC: 173.85
##
## Number of Fisher Scoring iterations: 5

anova(glm1,glm2, test="LRT")

## Analysis of Deviance Table
##
## Model 1: cbind(N_remain, N_float) ~ ChannelType + Flow + SpGr + Flow:SpGr
## Model 2: cbind(N_remain, N_float) ~ ChannelType + Flow + SpGr + Flow:SpGr +
## ChannelType:SpGr
## Resid. Df Resid. Dev Df Deviance Pr(>Chi)
## 1 29 89.434
## 2 28 86.110 1 3.3241 0.06827 .
## ---
## Signif. codes: 0 '***' 0.001 '**' 0.01 '*' 0.05 '.' 0.1 ' ' 1

# Coefficients:
#....
# Estimate Std. Error z value Pr(>|z|)
#ChannelTypeTest:SpGrlotic -0.7884 0.4362 -1.807 0.07071 .
# Indication that difference in number that remain (on the logistic scale) that remain between Control and Test is lower for lotic species than for lenthic species
anova(glm1,glm2, test="LRT")

## Analysis of Deviance Table
##
## Model 1: cbind(N_remain, N_float) ~ ChannelType + Flow + SpGr + Flow:SpGr
## Model 2: cbind(N_remain, N_float) ~ ChannelType + Flow + SpGr + Flow:SpGr +
## ChannelType:SpGr
## Resid. Df Resid. Dev Df Deviance Pr(>Chi)
## 1 29 89.434
## 2 28 86.110 1 3.3241 0.06827 .
## ---
## Signif. codes: 0 '***' 0.001 '**' 0.01 '*' 0.05 '.' 0.1 ' ' 1

summary(glm1)

##
## Call:
## glm(formula = cbind(N_remain, N_float) ~ ChannelType + Flow +
## SpGr + Flow:SpGr, family = binomial, data = Data, na.action = na.fail)
##
## Deviance Residuals:
## Min 1Q Median 3Q Max
## -3.8678 -1.1191 0.7191 1.2375 2.4597
##
## Coefficients:
## Estimate Std. Error z value Pr(>|z|)
## (Intercept) 2.9564 0.5137 5.755 8.65e-09 ***
## ChannelTypeTest 1.0873 0.2125 5.116 3.11e-07 ***
## Flowintermediate -3.2480 0.5467 -5.941 2.83e-09 ***
## Flowhigh -5.7213 0.5982 -9.563 < 2e-16 ***
## SpGrlotic 0.2984 0.7776 0.384 0.7012
## Flowintermediate:SpGrlotic 0.7182 0.8309 0.864 0.3874
## Flowhigh:SpGrlotic 1.8152 0.8548 2.124 0.0337 *
## ---
## Signif. codes: 0 '***' 0.001 '**' 0.01 '*' 0.05 '.' 0.1 ' ' 1
##
## (Dispersion parameter for binomial family taken to be 1)
##
## Null deviance: 450.081 on 35 degrees of freedom
## Residual deviance: 89.434 on 29 degrees of freedom
## AIC: 175.18
##
## Number of Fisher Scoring iterations: 5

# Coefficients:
#....
# Estimate Std. Error z value Pr(>|z|)
#Flowhigh:SpGrlotic 1.8152 0.8548 2.124 0.0337 *

# Evidence that difference in number that remain (on the logistic scale) between low and high flow is higher for lotic species than for lenthic species
# number that remain (on the logistic scale) = estimated probability of remaining in channel
